# Successful peritoneal dialysis for the end-stage kidney disease associated with Prader–Willi syndrome: a case report

**DOI:** 10.1007/s13730-019-00395-3

**Published:** 2019-04-08

**Authors:** Emi Anno, Keiichiro Hori, Ainori Hoshimoto, Makiko Harano, Sou Hagiwara, Kaori Oishi, Yoshinari Yokoyama, Yusuke Tsukamoto, Minoru Kubota

**Affiliations:** 1 Department of Nephrology, Itabashi Chuo Medical Center, 2-12-7 Azusawa Itabashi-ku, Tokyo, 174-0051 Japan; 2 Department of Nephrology, Ouji Hospital, 2-14-13 Ouji Kita-ku, Tokyo, 114-0002 Japan

**Keywords:** Prader–Willi syndrome (PWS), End-stage kidney disease (ESKD), Peritoneal dialysis (PD), Hybrid dialysis, Obesity, Diabetes mellitus

## Abstract

Prader–Willi Syndrome (PWS) is characterized by hyperphagia, severe obesity, and mental retardation from early childhood and occurs 1/10,000 to 1/15,000 live births in Japan. There is high prevalence of diabetes mellitus because of hyperphagia. The patient may sometimes face the necessity of renal replacement therapy (RRT) because of end-stage kidney disease (ESKD) caused by diabetes-associated kidney disease (DKD). Since mental retardation and extreme obesity usually prevent to introduce peritoneal dialysis (PD), hemodialysis (HD) has been the first choice of RRT. In this report, we experienced one case of patient with PWS suffering from ESKD due to DKD who started PD as an initial RRT and succeeded to continue for total of 40 months. The patient was 37-year-old man at the time of initiation of dialysis. PD was chosen for RRT because we suspected that he might have more technical difficulties for continuing HD. After several episodes of peritonitis, he successfully continues PD without peritonitis for next 27 months until the present time with good support by his family member. To our best knowledge, this is the first reported case of ESKD associated with PWS who was successfully treated with PD for long period.

## Introduction

Prader–Willi syndrome (PWS) is a complex neurodevelopmental genetic disorder with multiple cognitive, behavioral, and endocrine abnormalities caused by errors in genomic imprinting generally due to lack of paternally expressed genes from the 15q11–q13 region [1]. A shortened life expectancy is found in relationship to the level of intellectual disability primarily from complications of hyperphagia and obesity-related comorbidities [2]. Respiratory and other febrile illnesses are the most frequent causes of death in children, and obesity-related cardiovascular problems and gastric causes or sleep apnea are most frequent in adults. PWS patients become overweight in early childhood due to an insatiable appetite and, therefore, onset of DM is generally during adolescence at the earliest [3]. Prevalence of DM was reported 22% in 68 patients older than 13 years (age of youngest person with DM) in United Kingdom [3], whereas 26.2% in 65 Japanese patients older than 10 years (age of youngest person with DM) [4]. The type of DM was mostly non-insulin dependent in both cohorts [3, 4]. In this Japanese cohort, 5.9% showed proteinuria (> 300 mg/gCr) and 23.5% showed microalbuminuria (30–300 mg/gCr). Although this high prevalence of DM, this has been the only available data regarding diabetes-associated kidney disease (DKD) in PWS. End-stage kidney disease (ESKD) and its management by the renal replacement therapy (RRT) has been scarcely reported in PWS. However, it is easy to speculate that the RRT must be very difficult in this patient group because of the following problems: difficulty in creating and maintaining access for dialysis by either hemodialysis or peritoneal dialysis, patient behavioral and psychiatric disturbance to tolerate dialysis session and the short life expectancy and dependency on family members for life after all.

This is the first case report that demonstrates the feasibility of peritoneal dialysis in 37-year-old PWS patient.

## Case report

We report a case of a 37-year-old male with PWS who suffered from ESKD. At 3 years of age, he started overeating and developed obesity. At 5 years of age, PWS was suspected based on the patient’s body type, facial expression, and overeating. Dietary restriction was instructed; however, at 17 years of age, he was diagnosed with type 2 DM (T2DM). At 30 years of age, a diagnosis of PWS was confirmed by genetic testing. At age 32 years, he suffered from nephrotic syndrome, and diabetic retinopathy was diagnosed at the same time.

By June 2015, renal function had continuously deteriorated and his serum creatinine reached 8.9 mg/dL (eGFR at 5.0 mL/min/1.73 m^2^). Therefore, the patient was admitted to the hospital for initiating RRT. At admission, physical examination revealed: 150.8 cm height, 80.3 kg BW, BP 112/64 mmHg, HR 110/min, SO_2_ 99% (room air). He showed systemic edema and urine volume decreased to 800 mL/24 h. The patient’s IQ was inferred 40–60. Blood chemistry showed: TP 6.4 g/dL, albumin 2.4 g/dL, CRP 10.9 mg/dL, BUN 83 mg/dL, UA 6.4 mg/dL, Na 136 mEq/L, K 5.4 mEq/L, Cl 110 mEq/L, Ca 7.1 mg/dL, P 12.7 mg/dL and HCO_3_^−^ 14.7 mEq/L. Complete blood count was: WBC 16,770/μL, RBC 354 × 10^4^/μl, Hgb 8.1 g/dL, Ht 27.6%, MCV 78 fl, MCH 22.9 pg, MCHC 29.3% and Plt 54.2 × 10^4^/μl. Urinalysis showed massive proteinuria (4839 mg/24 h) without hematuria or pyuria. He had been receiving insulin injection 4 times a day. Random blood glucose level was 156 mg/dL and HbA1c was 6.8%.

After admission, restricted fluid intake, nutritional management, and temporary HD was performed 6 times for 2 weeks since massive edema prevented insertion of PD catheter. The reasons for choosing PD were as follows: vessels in his both forearms looked premature for creating A-V fistula at that time, suspecting intolerance in hemodialysis session for long hours and difficult cessation of fluid and food intake which are necessary for hemodialysis. Negative aspect of PD was specifically considered as a difficulty in maintaining sanitary in inlet of PD catheter. In either modality, family burden for supporting his dialysis was also considered.

**Fig. 1:**
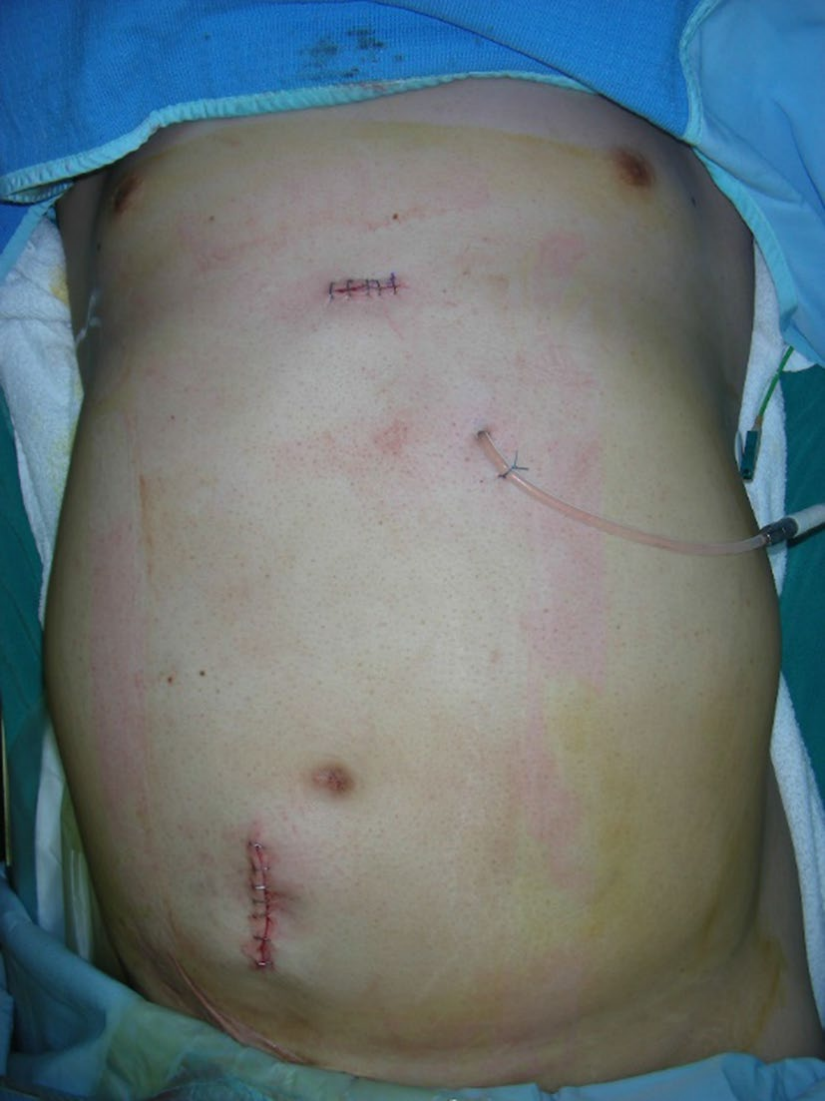
Insertion of PD catheter

**Fig. 2:**
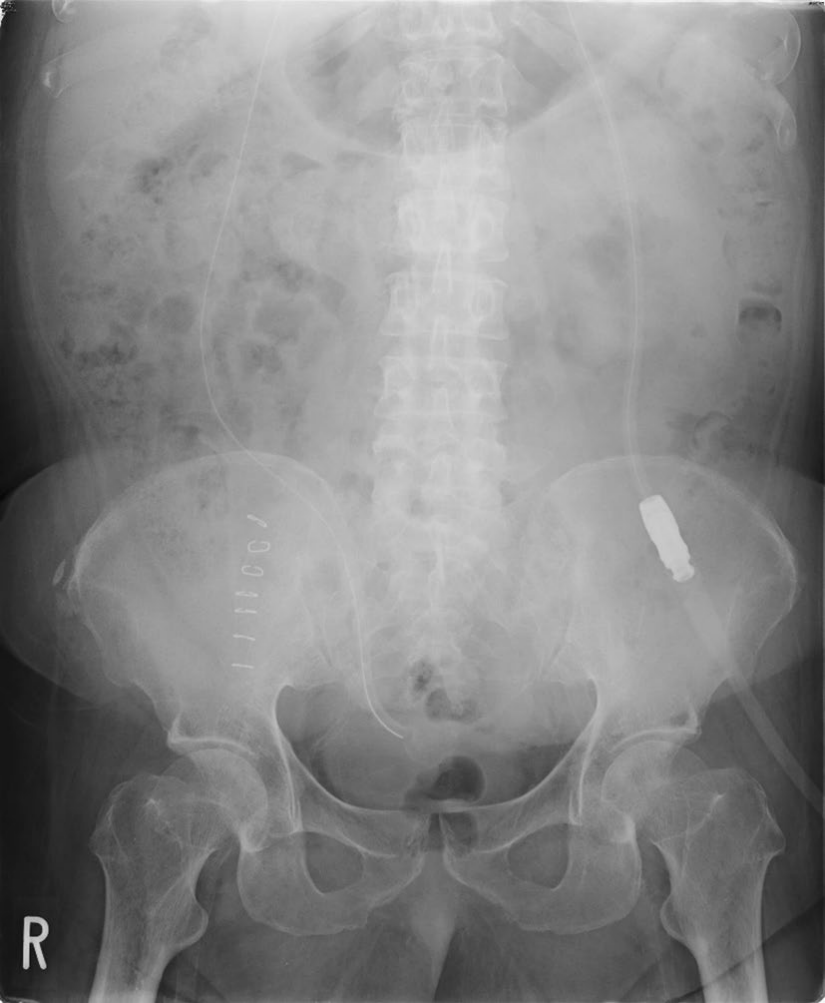
Abdomen X-ray after insertion of PD catheter

Substantially his body weight decreased to 64 kg (− 16 kg from admission; BMI 28.4 kg/m^2^) and a catheter for PD was inserted using the non-stylet method under general anesthesia combined with epidural anesthesia at the 14th day of admission. We selected the JBS-2 semi-long PD Catheter with the exit site placed in the left upper abdomen (Fig. 1). The catheter position was not ideally located initially but it functioned well for the following PD session after the surgery (Fig. 2). From the third postoperative day, nocturnal intermittent PD (NIPD) was started. Since he could not sufficiently understand the procedure of PD, instructions for PD as well as insulin self-injection were provided to his father. He was discharged at the day 35 of hospitalization.

**Fig. 3:**
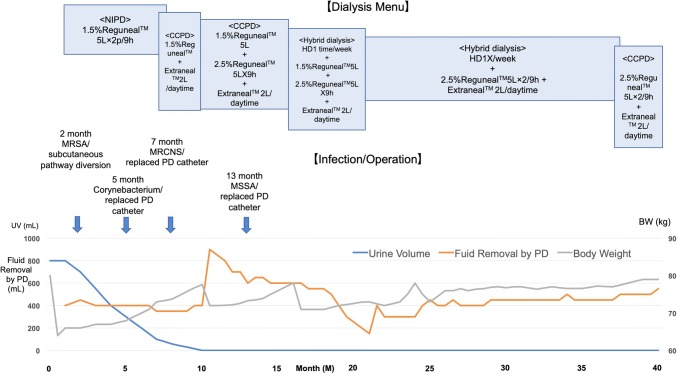
Clinical course of dialysis for 40 months. *NIPD* nocturnal intermittent peritoneal dialysis, *CCPD* continuous cycling peritoneal dialysis, *HD* hemodialysis, *MRSA* methicillin-resistant staphylococcus aureus, *MRCNS* methicillin-resistant coagulase-negative staphylococci, *MSSA* methicillin-sensitive staphylococcus aureus, *PD* peritoneal dialysis

At the 2nd, 5th, 7th, and 13th month after NIPD introduction, a subcutaneous tunnel infection occurred (Fig. 3). Since additional antibiotic therapy could not terminate infection, he underwent subcutaneous pathway diversion and PD catheter replacement for three times consequently. However, that was the last infection episode and infection-free PD was observed until the present time for next 27 months. With regard to the dialysis modality, NIPD was chosen first because he desired to continue his daytime job for handicapped person. At the 7th month after NIPD introduction, the treatment was switched to continuous cycling PD (CCPD) and 7.5% icodextrin solution (Extraneal™, Baxter, Tokyo, Japan) was added daytime because urine output gradually decreased, and ultrafiltration volume became insufficient. At the 10th month after NIPD introduction, glucose concentration in peritoneal dialysate increased from 1.5 to 2.5% (Reguneal™, Baxter, Tokyo, Japan). “Hybrid dialysis”, which combined daily PD with once-a-week HD, was started by creating A-V fistula at the 16th month after NIPD introduction. Owing to this “hybrid dialysis”, adequate ultrafiltration volume was maintained for following 17 months. Dialysis efficiency has been checked by blood tests and chest X-ray once a month and revealed adequate dialysis without major problems; accordingly, BUN level was below 50 mg/dL and serum potassium level below 5 mEq/L. Blood sugar was well maintained by daily intensive insulin therapy with oral dulaglutide initially and then this regimen was successfully replaced with once-weekly GLP-1 agonist associated with once a day ultralong-acting insulin finally. HbA1c had been maintained under 7% since NIPD introduction. His maximum body weight without overhydration reached to 71 kg (BMI 31.6 kg/m^2^) during this period. Finally, he lost his A-V fistula twice and returned to PD (CCPD) alone at the 38th month of NIPD introduction because of difficulty in creating another vascular access. For evaluating efficacy of PD, serum β2-microglobulin level was monitored every 2 months and revealed between 34 and 40 mg/L during the latest CCPD alone period.

## Discussion

PWS is considered the most commonly known genetic cause of life-threatening obesity in humans with a prevalence of 1 in 10,000 to 15,000 live births in Japan [5]. Symptoms are caused by hypothalamic dysfunction and generally include short stature, obesity due to hyperphagia, developmental delay, cognitive disability, behavioral problems such as short/hot temper, and hypersomnia. The degree of obesity is usually severe, and obesity or its complications could lead to death [6, 7]. In a survey conducted in the United States, the mean age of death in patients with PWS was 29.5 ± 16 years (range 2 months–67 years) due to mainly cardio-pulmonary failure (54% of all cause of death) [8]. Although PWS develops from infancy, the mortality rate before adulthood is only 30%, with 70% of patients growing into adults; therefore, this disease expects long-term management [8].

From the early stage, patients with PWS present severe obesity, and it often causes T2DM. The prevalence of T2DM in PWS is approximately 25% [4, 9]. One in four patients with PWS has been reported to develop T2DM at a mean age of 20 years, and the onset of T2DM carries an extremely high risk of complications in adult PWS [3]. Therefore, the progression to ESKD, similar to that observed in our patient, is assumed to be frequent in adult patients with PWS.

With regard to RRT, we examined applicability of each modality for this patient. Advantage of PD for such a patient is that can provide an opportunity of social rehabilitation. Disadvantage of PD for this type of patient is suspected as a difficulty in inserting catheter, weight and blood glucose management because of hyperphagia, and high risk of peritonitis caused by insufficient understanding of hygienic procedure. This patient and his family did not wish kidney transplantation either. For hemodialysis, vessels in his forearms looked immature for successful vascular access at first. As a matter of fact, an A-V fistula was once created successfully for “hybrid dialysis”, but it failed later possibly due to diabetic arteriopathy and severe obesity. Since the patient desired to continue his work on daytime, we decided that NIPD was mostly suitable for this patient.

The main caveats for PD management in this patient were difficulty in inserting PD catheter, infection control, diet and fluid management, blood glucose control and family support. Intraoperative manipulation was more difficult than usual because of extremely thick fat layer. Before operation, exact location of the inferior epigastric artery by echography was critical, and expert skill was required because the site of surgery was much deeper than ordinary procedure. However, Krezalek et al. reported that no significant difference was found in the actual rate of PD complications and the time for PD catheter failure between obese and non-obese patients [10]. When patients were divided based on those weighing ≥ 90 kg and those weighing < 90 kg, no significant difference was observed in the incidence of peritonitis [11].

During maintenance dialysis, this patient required his family support for treatment procedure. Infection occurred relatively often in this patient, with four episodes of peritonitis in first 13 months. Since this patient was not immunocompromised, we speculated that each episode of peritonitis was caused by tunnel infection due to his frequent play on his catheter. At this moment, withdrawal of PD or replacement of exit site to the back could be considered. However, we gave him another chance because the last interval between peritonitis was longer than previous one and eventually no new peritonitis has occurred for next 27 months until now. We supposed the reason for this long peritonitis-free interval was because that he had been accustomed to the presence of PD catheter and/or understanding the importance of PD catheter over time by education, then he touched his catheter less frequently than before.

With regard to ultrafiltration and body weight management, dietary restriction was not easy even with his family support at first. However, these disadvantages had been minimized using an icodextrin-containing dialysate, increasing dialysate concentration of glucose and later by the “hybrid dialysis”. His BMI increased after NIPD introduction. This weight gain was mainly due to recovery of his appetite after resolution of uremia. Then, his dry body weight had become stable even after increasing dialysate glucose. With regard to blood sugar control, this has not been too difficult in this patient and frequent insulin injection was successfully replaced with less frequent regimen finally. After all, availability of family support was a key for successful RRT in such a patient.

We are now seeking the way to create another vascular access because CCPD alone cannot provide sufficient ultrafiltration for him.

## Conclusion

For the end-stage kidney disease in PWS patient, PD can be a choice of modality if family member’s support is available.
